# Acupuncture and Multiple Sclerosis: A Review of the Evidence

**DOI:** 10.1155/2014/972935

**Published:** 2014-06-18

**Authors:** H. I. Karpatkin, D. Napolione, B. Siminovich-Blok

**Affiliations:** ^1^Hunter College, City University of New York, New York, NY 10010, USA; ^2^Department of Rehabilitation Medicine, New York University School of Medicine, Integrative Health Programs, Rusk Rehabilitation, New York University Langone Medical Center, New York, NY 10016, USA

## Abstract

Use of acupuncture to treat multiple sclerosis (MS) is fairly common, but little literature exists which studies its effectiveness. The purpose of this paper is to review the literature on the use of acupuncture to treat MS. A literature search resulted in twelve peer-reviewed articles on the subject that examined the use of acupuncture to treat MS related quality of life (QoL), fatigue, spasticity, and pain. The majority of the studies were poorly designed—without control, randomization, or blinding. Description of the subjects, interventions, and outcome measures as well as statistical analysis was often lacking or minimal. Although many of the studies suggested that acupuncture was successful in improving MS related symptoms, lack of statistical rigor and poor study design make it difficult to draw any conclusions about the true effectiveness of this intervention in the MS population. Further studies with more rigorous designs and analysis are needed before accurate claims can be made as to the effectiveness of acupuncture in this population.

## 1. Introduction

Multiple sclerosis (MS) is a disease affecting 300,000 people in the United States and 2.3 million worldwide [1]. It is characterized by autoimmune destruction of central nervous system (CNS) myelin, resulting in progressive loss of the function. Any CNS structure can be a potential target of MS, and, therefore, a great variety of symptoms are possible. Medical management had shown some success in limiting the frequency and intensity of disease activity, but only for persons with relapsing-remitting MS. Disease activity in patients with primary progressive or secondary progressive MS has not been shown to improve from medical intervention. Many persons with MS have also been dissatisfied with medical management due to perceptions that the medication has a range of unpleasant side effects. This has led many persons with MS to investigate the use of alternative therapies to treat their disease [2]. Acupuncture has been frequently mentioned as a nonpharmacologic means of managing the disease, with prevalence of its usage reported from 7.2 to 21% of the MS population [3, 4]. However, there have been very few studies that have investigated effectiveness of acupuncture use as a means of controlling disease activity or managing its symptoms. The use of acupuncture has been described in other neurologic conditions such as stroke [5], spinal cord injury [6], and Parkinson's disease [7], but there is very little research describing the use of acupuncture in MS. The purpose of this report is to review the studies on the use of acupuncture as a means of treating MS. In particular, the authors want to investigate if the literature published on the subject can support the use of acupuncture as a treatment for MS.

Since 1974, the literature of Chinese medicine describes MS as a “modern disease.” However, the earliest accounts of traditional Chinese medicine (TCM) refer to treatments of patients with MS associated patterns [8]. Understandably the definitions and pathology classifications used for MS in acupuncture have a very distinct language and logic that is separate and distinct from Western understandings of disease. As such, understanding the management of the disease and reporting its issues remain a challenge.

TCM is comprised of three different lines of therapy: herbal therapy, massage (Tui-Na and Gua-Sha), and acupuncture. Much of the research and what is seen being used therapeutically in the Western world appear to focus on acupuncture, but Tui-Na and Gua-Sha are frequently linked with acupuncture and, as a result, it is difficult to isolate the effects of acupuncture from these therapies. The basis for TCM diagnosis and treatment is based on a different understanding of illness and health from Western medicine and can appear unfamiliar. TCM asserts that the role of acupuncture is to analyze imbalances of distinct elements in the human body that can result in pathology [9]. Maciocia [10] states that MS is the result of a pathological accumulation of specific elements: dampness and phlegm. Dampness is said to have a quality that produces numbness and heaviness, while phlegm would produce double vision or poor memory. Sun [11] states that MS has five stages, characterized as (1) dampness/phlegm, (2) stomach and spleen deficiency (weakness), (3) liver and kidney deficiency (dizziness, vertigo), (4) liver and kidney yin deficiency with liver wind invasion (tremor), and (5) blood stasis (rigidity). Acupuncture treatments have to be both tailored to the correct disease stage and individualized to the patient's underlying constitution [9]. There are basic protocols proposed for those five different stages, which can be complemented with specific components designed to benefit each particular patient [10].

Given the individualized nature of acupuncture treatments and the multifactorial nature of MS, creating appropriate research protocols can be particularly challenging. As such, there is a compelling need to prove the efficacy of acupuncture in this population if its use is to be supported.

## 2. Materials and Methods

The following search terms were used: “Multiple sclerosis,” “Acupuncture,” “Traditional Chinese Medicine,” and “Demyelinating Disease.” We included all studies that used acupuncture to treat any symptom of MS or to evaluate disease activity. Studies that used acupuncture to treat animal models of MS were included. Only articles from peer-reviewed journals were accepted. Articles that did not differentiate acupuncture from TCM were excluded. Using these criteria, we comprehensively searched the following databases with no language restrictions: Ovid MEDLINE (1966 to June 2013), Cumulative Index to Nursing and Allied Health Literature (CINAHL, 1982 to June 2013), and Ovid Allied and Complementary Medicine (AMED, 1985 to June 2013). Acupuncture textbooks were also utilized.

## 3. Results

Fifteen articles were found that met our criteria. Of those articles, five examined the effect of acupuncture on quality of life (QoL) measures, three looked at the effects of acupuncture on MS fatigue, two examined the effects of acupuncture on MS spasticity, two examined the effect of acupuncture on MS pain, and three were animal studies. Several of the articles purported to study other symptoms such as bowel and bladder or gait but used exclusively QoL measures to evaluate outcomes. We therefore characterized these articles as QoL. The results are summarized in Tables 1 (study purpose, design, and subjects) and 2 (interventions, outcomes, results, and comments).

### 3.1. Acupuncture and QoL

Four studies were found that used QoL measures to evaluate the effects of acupuncture on MS.

Donnellan and Sharley [12] compared the effects of two types of acupuncture on QoL measures in fourteen persons with secondary progressive MS. Using a single-blind randomized control design, subjects received either Chinese acupuncture or minimal acupuncture. Chinese medical acupuncture was defined as the insertion of needles to a specified depth into particular acupuncture points that were chosen based on identification of traditional Chinese medical patterns corresponding with MS. Minimal acupuncture, the control intervention, was defined as the superficial insertion of needles into points located away from real acupuncture points and away from points important in the treatment of MS. Outcomes included the Multiple Sclerosis Impact Scale 29 (MSIS-29) and the Fatigue Severity Scale (FSS). After ten treatments over five weeks the minimal acupuncture group reported significantly greater improvement in the MSIS-29 psychological subscale compared with those receiving Chinese medical acupuncture in an intention-to-treat analysis (*P* < 0.04), with mean change in Chinese acupuncture group of 6.0 and in minimal acupuncture group of 23.0. No change was noted in the FSS. No measures of mobility or function were included, and there was no description of disease severity in the sample.

Spoerel et al. [13] described a three-month intervention where eight MS patients received nine to twenty-eight acupuncture treatments from two different practitioners. Although specific outcome measures were not described, some patients described a transient improvement in well-being. One patient was noted to have an improvement in spasticity during gait. No other improvements were noted. Descriptions of the subject characteristics, acupuncture protocols, or statistical analysis were not included in this brief report.

Hao et al. [14] presented a case report of a sixty-five-year-old male with a twenty-year history of MS. The patient was treated with Chinese scalp acupuncture once a week for ten weeks, then once a month for six more sessions. No outcome measures were reported, but the patient reported significant improvement in well-being, gait, balance, spasms, and incontinence. The authors concluded that scalp acupuncture has proven to have success in treating MS and other CNS damage as compared to other acupuncture modalities but provided no evidence for this statement.

Tjon Eng Soe et al. [15] examined the effects of electroacupuncture (EA) on nine MS patients with bladder dysfunction in a nonrandomized noncontrol pretest/posttest study. Patients received thirty minutes of EA once a week for ten weeks. Patients registered a voiding diary for three days and completed an incontinence-specific QoL (I-QoL) questionnaire both before and at the end of treatment. Daytime leakage episodes, urge frequency, and daytime and nocturnal voiding frequency over three days were also measured. After EA mean values for total I-QoL and all three subscores were higher than at baseline (0.05 < *P* < 0.10). Mean urge frequency decreased significantly by −2.21 from 3.89 to 1.68 times a day (*P* < 0.030) and mean number of daytime leaking episodes decreased by −0.78 from 1.18 to 0.40 (*P* < 0.041).

Quispe-Cabanillas et al. [16] evaluated the effect of the use of EA on QoL of patients with relapsing-remitting MS undergoing treatment with immunomodulators. Clinical status was monitored using the Expanded Disability Status Scale (EDSS). Thirty-one patients received thirty minutes of acupuncture once a week for six consecutive months in the randomized controlled study. EA improved various aspects of QoL, including pain reduction and depression. Self-report scales were found to be more sensitive to improvement than clinical measures.

### 3.2. Acupuncture and MS Fatigue

Fatigue is one of the most common MS symptoms [17] and its subjective and multifactorial nature makes effective pharmacologic treatment difficult. Acupuncture is purported to treat “blockage of energy meridians” in the body [9] and may therefore be an attractive treatment option for persons with MS who have experienced fatigue refractory to medical management. Three articles were found in which acupuncture was used to specifically treat MS fatigue.

McGuire [18] examined the effect of acupuncture on the fatigue of a fifty-year-old female with MS who received twenty minutes of acupuncture once a week for seven weeks. There were reports of improvement on three fatigue measures (Fatigue Severity Scale (FSS), Fatigue Impact Scale (FIS), and Fatigue Descriptive Scale (FDS)), but no statistical analysis was performed, nor was the subject described outside of the fatigue scales.

Foroughipour et al. [19] used a case series to evaluate the efficacy of acupuncture in treating fatigue in MS patients whose fatigue was resistant to the antifatigue medication Amantadine. Twenty MS patients received twelve sessions of acupuncture over two months following an unsuccessful trial of the antifatigue medication Amantadine. Five of the twenty patients recorded improved scores on the FSS. No further analysis was made of the subgroup that appeared to experience diminished fatigue following acupuncture.

Foell [20] hypothesized that fatigue and lack of coordination were central in origin and could be modified by providing intensive afferent input via EA in a twenty-five-year-old male with a nine-year history of RRMS. Duration was thirty minutes weekly for four weeks and then two more fortnightly sessions. Only descriptive subjective reports were recorded, including immediate change of sensation of his right leg and decreased feeling of “heaviness.” The patient also reported improved coordination and fewer slips and trips with effects lasting for eight months.

### 3.3. Acupuncture and Spasticity

Spasticity is a common finding in MS [21]. Treatment is largely pharmacologic, but patients have often found the medications to have unwanted side effects, especially sedation. Acupuncture may therefore be a viable alternative to medical management. While acupuncture has been found to be effective in treating spasticity in stroke patients [22], only one article was found which described its use in spasticity due to MS. Miller [23] examined the effects of acupuncture on spasticity in four women with MS: two ambulatory and two nonambulatory. Spasticity was measured using the Modified Ashworth Scale (MAS). The Barthel Index was also utilized to assess changes in ADL function. MAS scores were reported to improve in one patient; no improvement was seen in Barthel Index scores. No statistical analysis of the outcome measures was provided, nor was there a clear description of the sample.

### 3.4. Acupuncture and Pain

Pain is a relatively common finding in MS; patients reported to experience it 53–57% of the time over the course of their disease [24]. As is the case for spasticity, treatment is largely pharmacologic and can often result in lethargy. Additionally, MS pain is multifactorial, occurring for both primary and secondary causes. Acupuncture may provide a nonpharmacologic option for MS patients who experience pain. Two articles specified the use of acupuncture to treat pain associated with MS. Kopsky and Hesselink [25] described a case report where acupuncture was used along with PPAR-*α* agonist palmitoylethanolamide (PEA) to treat neuropathic pain in a sixty-one-year-old female with central neuropathic pain due to MS. The patient had scored a 4/10 on the Douleur Neuropathique 4 questionnaire. Treatment with acupuncture alone did not alleviate symptoms but decreased to 1/10 after the addition of PEA. The effect of PEA alone was not measured, and as such the effects of acupuncture in this study were not clear.

Tajik et al. [26] evaluated the efficacy of acupuncture in treating forty-nine MS patients with chronic pain using a case series pretest/posttest design. Subjects received biweekly acupuncture treatments for six months and noticed significant improvements in the Oswestry Disability Index (ODI). Use of concurrent pain medications was not reported, nor was discussion as to whether the pain was central or peripheral in nature.

### 3.5. Animal Models

A small number of articles evaluated the use of acupuncture on animal models of MS using rats with experimental autoimmune encephalitis (EAE). EAE is an experimentally induced disease that results in demyelination of the central nervous system and is widely used as an animal model to study MS in humans.

Liu et al. [27] found that rats with EAE treated with electroacupuncture had decreased disease severity, inhibited T-cell proliferation, and improved CD4+ T-cell balance as well as higher in vivo ACTH concentrations compared to control group rats. Huang et al. [28] found that demyelinated rats treated with electroacupuncture had increased production of oligodendrocyte precursor cells resulting in increased myelin formation. Spinal cord evoked potentials also improved in the experimental group. Liu et al. [29] examined the mechanism by which electroacupuncture effectively treated EAE, finding that the anti-inflammatory effects of electroacupuncture on EAE were related to *β*-endorphin production that balances the Thl/Th2 and Th17/Treg responses. These results suggest that *β*-endorphin could be an important component in the development of EA-based therapies used for the treatment of EAE.

## 4. Discussion

Despite what appears to be a large amount of anecdotal support for acupuncture in MS, there is a striking paucity of literature on the subject. The small number of studies on the subject are notable for small sample sizes, lack of reported statistical analysis, and insufficient or inconsistent descriptions of the sample. Many of the studies are case reports, and other studies have no randomization and little use of controls. Given these limitations, it is not surprising that the reports fail to show that acupuncture had a meaningful impact on MS.

Although the difficulties with these articles make recommendations for acupuncture for persons with MS difficult, it would be incorrect to assume that this suggests prohibiting its use in this population. Instead, it obviates the need for better-designed studies to be done on the topic. Studies with larger, better-described samples are needed. Study designs utilizing control groups, blinding, and randomization are needed, and those studies require appropriate statistical analysis. Appropriate outcome measures need to be carefully considered, as well as clearer definitions and descriptions of the treatment protocols used.

Interestingly, despite the fact that mobility loss is reported in 91% of all persons with MS [30], none of the studies specifically examined mobility measures such as gait or balance. This is of particular interest as nonpharmacologic interventions such as exercise have been shown to improve mobility loss in persons with MS [31]. If acupuncture can result in improvements in mobility in this population, it would be another attractive option for intervention for patients looking for effective therapy that did not necessitate further use of medication.

It is notable that, of the human studies that did adequate jobs of reporting outcomes, few of the outcomes were positive. Although it is tempting to conclude that this is due to the fact that acupuncture was not an effective treatment, there are many other possible reasons as to why this may have occurred. First, it may be that the measures used were not sufficiently sensitive to changes that occurred as a result of treatment. The effects of acupuncture may not be of such a nature that the impact scales commonly used to assess change in MS are insufficient. This suggests that future studies should utilize different or additional outcome scales. It may also have been the case that the period of measurement or intervention was simply too short or of too small a volume to result in meaningful changes. It is possible that if the acupuncture intervention was carried out over a longer period of time or if the measurements were taken in an ongoing manner over this period, more of a difference might have been seen. Future studies might utilize both a longer period of intervention and measurements taken in an ongoing manner as opposed to a pretest/posttest design.

## 5. Conclusion

Although there is evidence of extensive use of acupuncture in treating MS, there is a striking paucity of literature available to describe its use or its efficacy. Extensive methodological flaws mar the few studies that do exist. Despite this, practitioners should not assume that acupuncture is not effective in this population but rather that the literature is insufficient to make claims either for or against its use. Future studies that might address some of the methodological flaws need to be done before more definitive statements as to the effectiveness of acupuncture in MS can be made.

## Figures and Tables

**Table 1:** 

Study	Purpose	Design	Subjects
Donnellan and Sharley (2008) [12]	To compare the effect of two types of acupuncture on the quality of life of individuals with secondary progressive multiple sclerosis and provide preliminary evidence regarding the safety of this intervention for this population.	RCT	*N* = 14; secondary progressive MS

Spoerel et al. (1974) [13]	Evaluate the success of application of acupuncture on MS.	Case study series (8 patients)	*n* = 8

Hao et al. (2013) [14]	Assess Chinese scalp acupuncture effectiveness for MS.	Case study	*n* = 1; 65-year-old male; MS for 20 years

Tjon Eng Soe et al. (2009) [15]	Assess whether electroacupuncture diminishes voiding symptoms and improves QoL in MS patients with overactive bladder.	Nonrandomized noncontrol pretest/posttest	*n* = 10; 9 males and 1 female; 6 SPMS and 3 RRMS. EDSS: 2.0–6.5, and 6 with a score of 6.0

Quispe-Cabanillas et al. (2012) [16]	Evaluate the effect of the use of acupuncture on QoL of patients with RRMS undergoing treatment with immunomodulators.	Double-blind RCT	*n* = 31; RRMS, undergoing treatment with immunomodulators randomly distributed into sex-stratified experimental and placebo groups

McGuire (2003) [18]	Examine effect of acupuncture on fatigue in a patient with MS.	Case study	*n* = 1; 50-year-old female

Foroughipour et al. (2013) [19]	Evaluate the efficacy of acupuncture in fatigue in patients with MS whose symptoms are resistant to conventional drug therapies.	Case series; pretest/ posttest	*n* = 20; individuals with MS nonresponsive to 2 months of Amantadine treatment (FSS did not drop below 30); mean FSS: 48 ± 8.6; mean age: 33.85 ± 10.85 years; 75% of women, EDSS > 4

Foell (2011) [20]	Highlight a single case of “off-label” use of acupuncture and its effects on symptoms.	Case report	*n* = 1; 25-year-old male with RRMS for 9 years

Miller (1996) [23]	Evaluate the effectiveness of acupuncture in MS spasticity.	Nonrandomized, control group and single-blind ABA	*n* = 4; 4 women with MS: 2 ambulatory and 2 nonambulatory; 38–54 years old; all receiving medication, physiotherapy, and hydrotherapy to varying degrees

Kopsky and Hesselink (2012) [25]	Assess a multimodal approach for MS acupuncture + PEA.	Case study	*n* = 1; 61-year-old; chronic central neuropathic pain

Tajik et al. (2012) [26]	To evaluate the efficacy of acupuncture in the treatment of chronic pain and fatigue in patients with MS.	Case series; pretest/ posttest	*n* = 49; MS diagnosis, referred to alternative medicine

Liu et al. (2010) [27]	To examine whether electroacupuncture could provide protection against experimental autoimmune encephalitis (EAE) in a rat model for MS. In addition, to assess the effects of acupuncture on EAE CD4+ Th cell profiles.	Pretest/posttest with 4 treatment groups	8-week-old female Lewis rats

Huang et al. (2011) [28]	To analyze if governor vessel (GV) electroacupuncture (EA) could efficiently promote increase in cell number and differentiation of OPCs into oligodendrocytes, remyelination, and functional recovery in the demyelinated spinal cord.	Animal (rat), randomized group assignment (6)	*N* = 70; dorsal laminectomies were performed at the T9/T10 level; ethidium bromide (EB) was injected to induce demyelination in the EB injection group

Liu et al. (2013) [29]	To characterize the effects of electroacupuncture on rats with experimental autoimmune encephalomyelitis.	Pretest/posttest with 4 treatment groups	8-week-old female Lewis rats

**Table 2:** 

Study	Intervention	Outcome measures	Results	Conclusion/comments
Donnellan and Sharley (2008) [12]	10 acupuncture treatments of 25-minute duration over 5 consecutive weeks; 2 groups: minimal acupuncture group received acupuncture into very superficial levels and Chinese acupuncture group received acupuncture at true acupuncture site and depth.	Multiple Sclerosis Impact Scale 29, Fatigue Severity Scale, and Health Questionnaire 12	Participants receiving minimal acupuncture demonstrated statistically significant greater improvement in the Multiple Sclerosis Impact Scale 29 psychological subscale compared with those receiving Chinese medical acupuncture in an intention-to-treat analysis (*P* < 0.004), with mean change in Chinese acupuncture group of 6.0 (SD 13.9) and in minimal acupuncture group of 23.0 (SD 21.0).	Minimal acupuncture resulted in greater improvement of Multiple Sclerosis Impact Scale 29 psychological subscale compared with Chinese medical acupuncture. No adverse effects noted.

Spoerel et al. (1974) [13]	From 7 to 28 treatments over a period of 3 months.	QoL (functional improvement and improved sensation of “well-being”)	Improvements noted in spasticity in 1 patient (ambulatory). No statistical analysis shown.	After reviewing 8 patients, they concluded that there is no evidence that acupuncture has any permanent effect on multiple sclerosis. In some patients there is a reported transient functional improvement and a feeling of well-being.

Hao et al. (2013) [14]	Once a week for 10 weeks and then once a month for 6 sessions. Chinese scalp areas were motor, sensory, foot motor and sensory, balance, hearing, dizziness, and tremor area. Ear point was shen men.	QoL (improvement in his dizziness, balance, stiffness, and weakness in his legs)	Scalp acupuncture in this patient has proven to have superior success in treating MS and other central nervous system damage as compared to other acupuncture modalities.	Future study is needed to investigate the mechanisms underlying acupuncture's effect on the central nervous system dysfunctions in patients with MS.

Tjon Eng Soe et al. (2009) [15]	Electroacupuncture to posterior border of tibia and medial edge of foot bilaterally for 30 minutes 1x/week for 10 weeks.	Recorded daytime leakage episodes, urge frequency, and daytime and nocturnal voiding frequency over 3 days. Also registered a voiding diary for 3 days and completed the validated incontinence-specific QoL (I-QoL) questionnaire before and at end of treatment	Mean urge frequency decreased significantly by −2.21 from 3.89 to 1.68 times a day (**P** **=** 0.030). Mean number of daytime leaking episodes by −0.78 from 1.18 to 0.40 (**P** **=** 0.041) of the decrease in mean urge frequency was in the same range as that seen in MS patients treated with tolterodine. After EA mean values for total I-QoL and all three subscores were higher than at baseline (0.05 **<** **P** **<** 0.10). The mean difference was comparable with that of PTNS.	Electroacupuncture may provide a useful role in treatment. Results justify a larger, randomized study.

Quispe-Cabanillas et al. (2012) [16]	30 minutes of electroacupuncture (either true or sham) once a week for six consecutive weeks.	Disability: EDSS, Pain: VAS, QoL: functional assessment of MS	Electroacupuncture improved various aspects of QoL, including reduction in pain and depression. Self-report scales were more sensitive to improvement than clinical measures. Evaluated using SAS system, Mann-Whitney *U* test, and ANOVA.	Evidence that electroacupuncture can significantly improve QoL of this population suggests that routine use of a self-report scale evaluating QoL should be included in regular clinical evaluations in order to detect change more accurately.

McGuire (2003) [18]	20-minute sessions 1x/week for 7 weeks. Points located on both upper and lower limbs, along the spleen, stomach, gallbladder, kidney, urinary bladder, and large intestine.	Fatigue: Fatigue Impact Scale, Fatigue Severity Scale, and Fatigue Descriptive Scale	Improvements in all fatigue measures, and no statistical analysis was done.	Subjectively, subject reported an increase in energy levels, the ability to take on more activities, being more able to be part of conversations, and being able to socialize more frequently.

Foroughipour et al. (2013) [19]	Total of 12 sessions of 30-minute duration of acupuncture were conducted every other day for 4 weeks.	Fatigue: FSS	5/20 (25%) demonstrated decreased FSS scores to <30 after 12 acupuncture sessions. Mean reduction of all 20 patients: 20.6 ± 7.2.	Acupuncture appears to be associated with benefits for a portion of patients with fatigue who are resistant to conventional drugs, such as Amantadine, and justifies further research.

Foell (2011) [20]	Intramuscular stimulation with electroacupuncture in tibialis anterior and flexor hallucis longus bilaterally. Duration was 30 min weekly for 4 weeks and then two more fortnightly sessions.	Descriptive subjective reports only. Reported immediate change of sensation of his right leg and decreased feeling of “heaviness.” Reported improved coordination and fewer slips and trips. Stated that effects lasted for 8 months	Nonspecific narrative of improvements in gait and balance.	Researchers reported an improvement in overall attitude towards the illness. Following the study, this subject was able to take up regular exercise and reported an increase in his “sense of control” over his life.

Miller (1996) [23]	Test group treated with short course of acupuncture consisting of 4 treatments administered once every 4 days.	(1) Spasticity: the Ashworth Scale; (2) functional ability: the Standard Barthel Activities of Daily Living Index	One subject demonstrated improved Barthel Index score, and 1 subject demonstrated improved Ashworth score.	Three patients reported subjective improvements after acupuncture treatments, stating that they were “more able to cope with the problem.”

Kopsky and Hesselink (2012) [25]	In 2009, 4 long acupuncture needles were placed in medial and lateral borders of the radius near the wrist for 30 min. In 2011 PEA was added (PEA levels were adjusted a few times, depending on pain scale).	QoL and pain levels were monitored. 11-point Numerical Rating Scale (NRS)	Pain reduction after a multimodal approach.	PEA reduces pain via the natural modulation pathways and acupuncture sessions can enhance the analgesic effects, when the analgesic response waivers.

Tajik et al. (2012) [26]	Acupuncture treatment biweekly for 6 months.	Pain and fatigue: Oswestry Disability Index	Mean ODI before: 41.16 ± 3.74, after: 33.59 ± 5.14. Additionally, all 10 measures in ODI decreased significantly after 6 months of treatment.	Fatigue and chronic pain are subjective and multifactorial. Further studies with larger sample sizes and adjustment for EDSS scores required before acupuncture can be suggested as a complementary treatment.

Liu et al. (2010) [27]	Electroacupuncture daily for 30 min for a period of 7, 14, and 21 days.	Spinal cord samples, lymphocyte cultures, intracellular and extracellular cytokine detection, and plasma and hypothalamus ACTH concentrations were all analyzed posthumously	Rats with EAE treated with electroacupuncture had decreased disease severity, inhibited T-cell proliferation, and improved CD4+ Th cell balance as well as higher in vivo ACTH concentrations compared to control group rats.	This is the first study to describe the physiological benefits of acupuncture on rat models.

Huang et al. (2011) [28]	Electroacupuncture (EA). EA was administered every other day for 26 days, starting from the third day after surgery. The intensity was adjusted to induce slight twitch of the hindlimbs, for 20 min in the EA treatment group. EA stimulation was performed at two acupoints in governor vessel: Jizhong (GV6) and Zhiyang (GV9).	(1) Neurotrophin-3 (NT-3) level, (2) number of NG2 positive OPCs between neurofilament (NF) positive nerve fibres, (3) number of adenomatous polyposis coli- (APC-) positive oligodendrocytes of newly formed myelin, (4) behavioral tests, and (5) spinal cord evoked potential detection functional recovery	EA treatment can promote NT-3 expression, increase of the cell number and differentiation of endogenous OPCs and remyelination in the demyelinated spinal cord as well as the functional improvement of demyelinated spinal cord.	The results suggest that EA treatment could effectively promote the functional recovery after the demyelinating lesion. EA would be a new and safe therapeutic strategy for treatment of MS patients.

Liu et al. (2013) [29]	Electroacupuncture daily for 21 days.	In vivo and in vitro analysis of apoptosis, lymphocytes, and T-cell proliferation	Anti-inflammatory effects of electroacupuncture on EAE were related to *β*-endorphin production that balances the Thl/Th2 and Th17/Treg responses.	*β*-Endorphin could be an important component in the development of EA-based therapies used for the treatment of EAE.
